# LONELINESS AND SELF-RATED DAILY SLEEP QUALITY: RESULTS FROM THE EINSTEIN AGING STUDY

**DOI:** 10.1093/geroni/igac059.2925

**Published:** 2022-12-20

**Authors:** Jee eun Kang, Orfeu Buxton, Jinshil Hyun, Martin Sliwinski

**Affiliations:** The Pennsylvania State University, University Park, Pennsylvania, United States; The Pennsylvania State University, University Park, Pennsylvania, United States; Albert Einstein College of Medicine, Bronx, New York, United States; The Pennsylvania State University, University Park, Pennsylvania, United States

## Abstract

Poor sleep quality is a common problem and has profound physical and mental effects on older adults. Loneliness, another common and harmful condition for older adults, is associated with heightened levels of vigilance and feeling unsafe, which may contribute to poor sleep. Although some studies show that trait loneliness is related to compromised sleep, there are few studies examining whether and how day-to-day variations in loneliness relate to sleep quality. The current study aimed to examine within- and between-person associations of loneliness and daily self-rated sleep quality using ecological momentary assessment (EMA) approach. Community-dwelling participants (n=317, Mage=77 (range=70–90), 56% white) reported loneliness and self-rated sleep quality during a 14-day EMA study via mobile phones. Trait loneliness was measured once prior to EMA. Unexpectedly, multilevel modeling showed that the amount of loneliness experienced on a given day did not predict sleep quality, however lower levels of prior night sleep quality predicted elevated levels of loneliness (p < .01). Regarding the between-person association, individual differences in trait loneliness were not related to sleep quality, but differences in average levels of EMA loneliness were related to sleep quality, such that people who felt higher levels of loneliness on average during the 14 days reported worse quality of sleep during that time period than others (p < .0001). These results suggest that worse sleep quality may be a factor that increases loneliness, and may not be a consequence of loneliness. Potential benefits of using EMA loneliness in research of daily sleep quality in older adults will be discussed.

